# Temporal trends of sex differences for COVID-19 infection, hospitalisation, severe disease, intensive care unit (ICU) admission and death: a meta-analysis of 229 studies covering over 10M patients

**DOI:** 10.12688/f1000research.74645.1

**Published:** 2022-01-05

**Authors:** Bart G. Pijls, Shahab Jolani, Anique Atherley, Janna I.R. Dijkstra, Gregor H.L. Franssen, Stevie Hendriks, Evan Yi-Wen Yu, Saurabh Zalpuri, Anke Richters, Maurice P. Zeegers

**Affiliations:** 1 Orthopaedics, Leiden University Medical Center, Leiden, The Netherlands; 2 Department of Methodology and Statistics, Care and Public Health Research Institute (CAPHRI), Maastricht University, Maastricht, The Netherlands; 3 School of Health Professions Education, Department of Educational Research and Development, Maastricht University, Maastricht, The Netherlands; 4 Vrije Universiteit Medical Centre, Amsterdam, The Netherlands; 5 Maastricht University Library, Maastricht University, Maastricht, The Netherlands; 6 School of Mental Health and Neuroscience (MHeNS), Maastricht University, Maastricht, The Netherlands; 7 Department of Complex Genetics and Epidemiology, School of Nutrition and Translational Research in Metabolism, Maastricht University, Maastricht, The Netherlands; 8 Key Laboratory of Environmental Medicine and Engineering of Ministry of Education, Department of Epidemiology & Biostatistics, School of Public Health, Southeast University, Nanjing, China; 9 Real World Evidence, UCBPharma, Breda, The Netherlands; 10 Department of Research and Development, The Netherlands Comprehensive Cancer Organisation, Utrecht, The Netherlands; 11 Department of Epidemiology, School of Nutrition and Translational Research in Metabolism, Care and Public Health Research Institute, Maastricht University, Maastricht, The Netherlands

**Keywords:** COVID-19, sex-differences, male, femal, mortality, ICU admission, infection, severity

## Abstract

**Background:** This review aims to investigate the association of sex with the risk of multiple COVID-19 health outcomes, ranging from infection to death.

**Methods:** Pubmed and Embase were searched through September 2020. We considered studies reporting sex and coronavirus disease 2019 (COVID-19) outcomes. Qualitative and quantitative data were extracted using standardised electronic data extraction forms with the assessment of Newcastle Ottawa Scale for risk of bias. Pooled trends in infection, hospitalization, severity, intensive care unit (ICU) admission and death rate were calculated separately for men and women and subsequently random-effects meta-analyses on relative risks (RR) for sex was performed.

**Results:** Of 10,160 titles, 229 studies comprising 10,417,452 patients were included in the analyses. Methodological quality of the included studies was high (6.9 out of 9). Men had a higher risk for infection with COVID-19 than women (RR = 1.14, 95%CI: 1.07 to 1.21). When infected, they also had a higher risk for hospitalization (RR = 1.33, 95%CI: 1.27 to 1.41), higher risk for severe COVID-19 (RR = 1.22, 95%CI: 1.17 to 1.27), higher need for Intensive Care (RR = 1.41, 95%CI: 1.28 to 1.55), and higher risk of death (RR = 1.35, 95%CI: 1.28 to 1.43). Within the period studied, the RR for infection and severity increased for men compared to women, while the RR for mortality decreased for men compared to women.

**Conclusions:** Meta-analyses on 229 studies comprising over 10 million patients showed that men have a higher risk for COVID-19 infection, hospitalization, disease severity, ICU admission and death. The relative risks of infection, disease severity and death for men versus women showed temporal trends with lower relative risks for infection and severity of disease and higher relative risk for death at the beginning of the pandemic compared to the end of our inclusion period.

**PROSPERO registration:** CRD42020180085 (20/04/2020)

## Introduction

The role of sex has been a topic of interest in many coronavirus disease 2019 (COVID-19) studies, with many countries reporting higher case fatality rates among men than women ^1^. There is, however, considerable variability among estimated effects of sex across countries for several relevant COVID-19 outcomes, including death. A recent systematic review from our group summarized literature from the earliest pandemic phase to show the impact of age and sex on commonly reported COVID-19 related outcomes ^2^. We showed that men were observed to have a higher risk of confirmed COVID-19 infection among those tested, and more often had severe COVID-19 disease, required intensive care unit (ICU) admission and had a fatal outcome when hospitalized with COVID-19 ^2^, a finding that other researchers ^3, 4^ have confirmed and has led to speculation on biological mechanisms ^5, 6^. However, almost all studies from previous systematic reviews were based on the early phase of the pandemic where testing was less commonly available, treatments were not yet evidence-based and mortality rates were high.

There are some indications that ICU and mortality rates have now decreased ^7– 9^, raising the question of whether men are still at increased risk in different severity stages ranging from infection to death now that substantially more data is available.

Here we present a systematic review and meta-analysis on the association between sex and risk of COVID-19 for infection, hospitalization, disease severity, ICU admission and mortality. Studies in this review cover all continents with the exception of Antarctica, covering study populations from a large timeframe of the COVID-19 pandemic. This should provide a more nuanced insight into the association of sex with relevant COVID-19 outcomes and temporal variety of the association.

## Methods

The reporting of this systematic review and meta-analysis is in accordance with the Preferred Reporting Items for Systematic reviews and Meta-Analyses (PRISMA) statement ^10^ and a protocol has been registered *a priori* at the Prospero registry (CRD42020180085, 20 ^th^ April 2020) ^11^. The review aimed to quantify the relative risk of men versus women on five commonly reported COVID-19 related outcomes, specifically (see also Table 1): 1) confirmed COVID-19 infection among the general population; 2) hospitalization among patients with a confirmed COVID-19 infection; 3) severe (clinically/radiologically) COVID-19 among hospitalized patients with a confirmed COVID-19 infection; 4) ICU admission among hospitalized patients with a confirmed COVID-19 infection; and 5) death among hospitalized patients with a confirmed COVID-19 infection.

**Table 1.** Study structure. COVID-19=coronavirus disease 2019; ICU=intensive care unit.

Severity stage	Case	control	population
1 Infection	Test positive	Test negative	General population
2 Hospitalization	Hospitalized	Not hospitalized	Confirmed COVID-19 cases
3 Severe symptoms (clinically or radiologically)	Severe symptoms	Non-severe symptoms	Hospitalised COVID-19 cases
4 ICU admittance	Admitted to ICU	Not admitted to ICU	Hospitalised COVID-19 cases
5 death	Death	alive	Hospitalised COVID-19 cases

### Data sources and searches

The search strategy was devised with a specialised librarian (GF) and the following databases were searched from December 1st 2019 up to an including September 17th 2020: Medline via PubMed and EMBASE. We designed the search strategy to be sensitive and reproducible. The term COVID-19 was elaborated in combinations of controlled vocabulary and free text terms. See Appendix I in the *Extended data* ^12^ for the full search strategy. Studies reported in languages spoken by the research team were eligible: English, Dutch, German, French, Spanish, and Russian.

### Study selection

Initial screening on the basis of title and abstract of eligible studies was performed by allocating subsets of the search results to all members of the review team. When the information in the abstract did not suffice or where there was any doubt, the studies remained potentially eligible. The full text of potentially eligible studies was evaluated by a member of the review team (MZ, BP, AR, AA, JD, SH, EY and SZ). All records identified through the searches were collected in an electronic reference database and subjected to the following inclusion and exclusion criteria: the study had to focus on humans with COVID-19 or severe acute respiratory syndrome coronavirus 2 (SARS-CoV-2) infections providing, or potentially providing, sufficient information to calculate relative risk for our pre-specified associations ( Table 1). Studies were excluded when there was no data on controls, when the study focussed on a specific population (e.g., health care workers, paediatric patients), when the study quality score (see next paragraph) was less than 5 out of 9 and when patients were admitted to hospital for different indications than for COVID-19 (e.g., kidney transplant patients, patients with fractured bones).

### Data extraction and quality assessment

A reviewer team (MZ, BP, AR, AA, JD, SH, EY and SZ) from the review team extracted data from included studies regarding patient demographics, study characteristics, and the severity stages of COVID-19 including infection, hospitalization, severity, ICU admission, and death. To ensure data quality, a reviewer team (MZ, BP, AR, AA, JD, SH, EY and SZ) from the review team other than the one who initially included the paper, confirmed the inclusions and data extraction for 20 randomly selected papers. Additionally, one of the reviewers (AR) checked the inclusion and data extraction of all studies that were potential outliers in terms of absolute rate of the outcome or RR as visually identified on the forest plots. Risk of bias of the included studies was appraised using the Newcastle Ottawa Scale (NOS) ^13^.

### Data synthesis and analysis

We used relative risks (RR) to assess the association between each severity stage (i.e., infection, hospitalization, severity, ICU admission, and death) and sex and performed a random-effects meta-analysis to determine the pooled effect sizes with corresponding 95% confidence intervals and 95% prediction intervals ^14^. The amount of statistical heterogeneity was assessed through visual inspection of the forest plots and by calculating I² statistics ^15^. If data allowed, we explored potential sources of statistical heterogeneity when I ^2^ was above 40% (1) through subgroup analyses and (2) with random-effects meta-regression analyses on pre-defined factors, including geographical region, study quality, study size, days into the pandemic based on study start and end date, publication date, diagnostic modality (e.g., polymerase chain reaction [PCR] test, computerized tomography [CT] signs, clinical symptoms and their combinations that led to the diagnosis of COVID-19), and clinical setting (e.g., nursing home, home, hospital, general practitioner [GP] cohort).

To assess publication bias, we constructed funnel plots for visual inspection and statistically tested potential asymmetry using the Egger and Harbord test ^16, 17^. In case of asymmetry, a trim-and-fill method and cumulative meta-analyses was used to explore the magnitude and direction of publication bias.

***Patient and public involvement statement*.** This systematic review and meta-analysis is part of the World Health Organization (WHO) Evidence Collaborative on COVID-19 answering on of their rapid review priority questions on risk factors for infection and disease severity. Patients were not involved.

## Results

### Study selection

**Figure 1.**
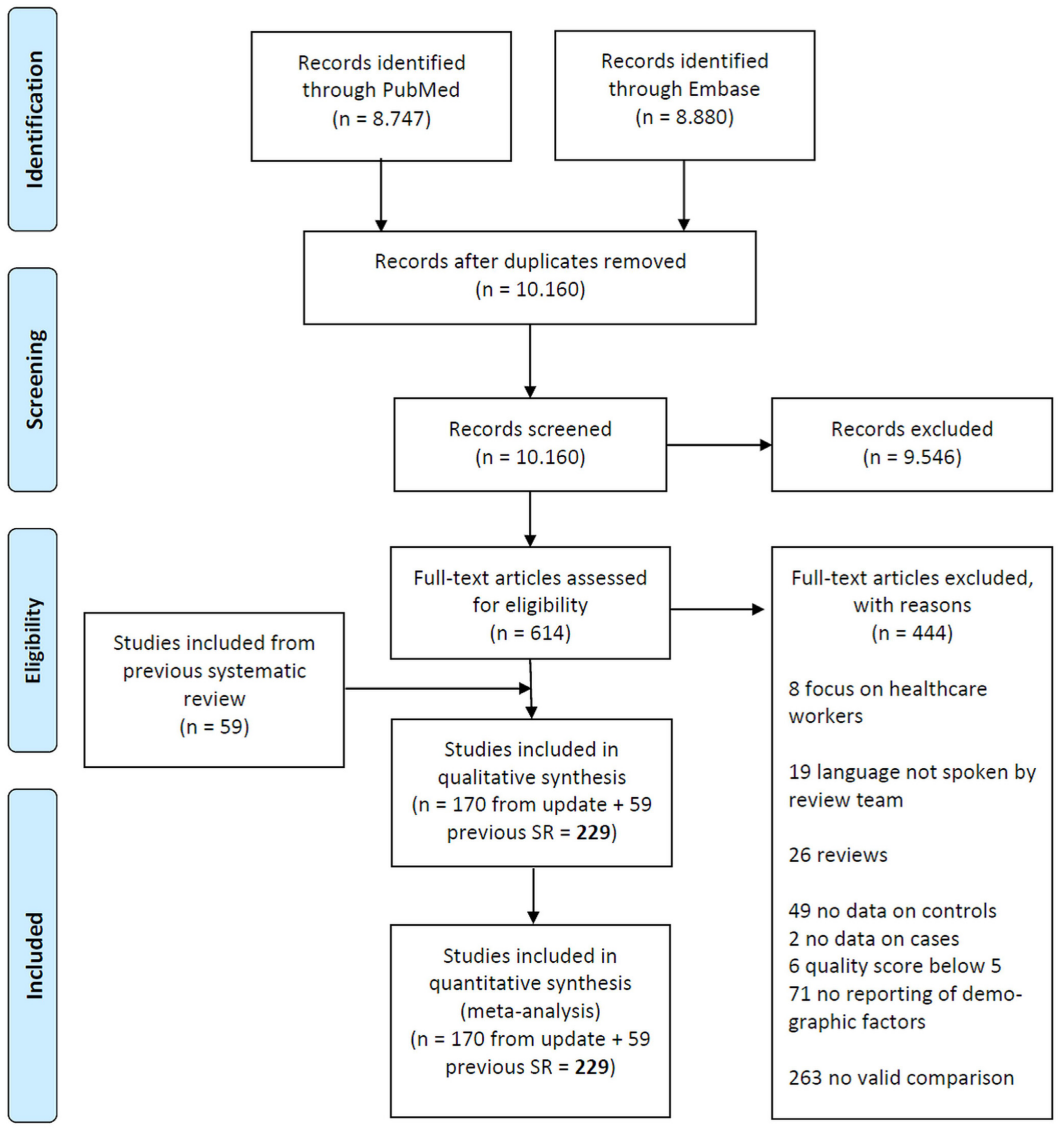
PRISMA flow chart showing study selection.

The literature search yielded 10,160 unique hits of which 614 studies were eligible after screening titles and abstracts. From these eligible studies, we excluded 444 studies: 8 focused on healthcare workers only, 19 were in languages not spoken by the research team; 26 were reviews; 49 had no data on controls; 2 had no data on cases; 6 scored below 5 on the New Castle Ottawa Scale; 71 did not report or evaluate sex differences; and 263 made no valid comparison between men and women. This left 170 studies and together with 59 studies from the previous systematic review the total number of included studies was 229 covering a total of 10,417,452 patients. Details of the study selection and included studies are given in Figure 1 (PRIMSA flow chart) and Appendix II in the *Extended data* ^12^.

### Study characteristics

Of the included studies, 94 were from China, 42 from the United States, 18 from Italy, 11 from the United Kingdom, 8 from South-Korea, 6 from Germany, 6 from Spain, 6 from Turkey, 4 from Brazil, 4 from Mexico, 3 from Denmark, 3 from Iran, 3 from Israel, 2 from Austria, 2 from India, 2 from Switzerland, 2 from Thailand, and 1 from each of the following countries: Argentina, Australia, Belgium, France, Honduras, Indonesia, Japan, Kuwait, Netherlands, Poland, Singapore, South Africa, and Sweden. Study size ranged from 21 to more than 8,000,000 individuals. The included studies recruited individuals in the period between December 1 ^st^ 2019 and August 19 ^th^ 2020. Data of individual studies, organized by exposure and outcome, are available as *Underlying data*.

### Risk of bias

The methodological quality of the included papers was relatively high with an average of 6.9 out of 9, as measured with the Newcastle Ottawa Scale (NOS). Details of NOS items for individual studies, organized by exposure and outcome are available as *Underlying data*.

### Outcomes

Meta-analyses of the primary outcomes for the risk factor sex revealed differences among men and women ^18^. An overview of the pooled results from random-effects meta-analyses for the risk factor sex can be found in Table 2. There was an unambiguous association between each stage of disease severity and sex with men having a higher risk of infection, hospitalization, disease severity, ICU admission and death than women.

**Table 2.** Summary of data synthesis.

Exposure	Outcome	Number of studies	Number of patients	Pooled estimate (RR)	95% CI	95% PI	Heterogeneity (I ^2^)
Sex (male vs female)	Infection	41		1.14	1.07 to 1.21	0.79 to 1.63	98.6 %
	hospitalization	22		1.33	1.27 to 1.41	1.06 to 1.68	90.9%
	Severe disease	77		1.22	1.17 to 1.27	1.02 to 1.45	46.7%
	ICU	48		1.41	1.28 to 1.55	0.83 to 2.40	80.6%
	Death	91		1.35	1.28 to 1.43	0.90 to 2.03	82.1%

RR = relative risk; 95% CI = 95% confidence interval; 95% PI = 95% prediction interval; ICU=intensive care unit.

**1. Risk of infection**

**Figure 2.**
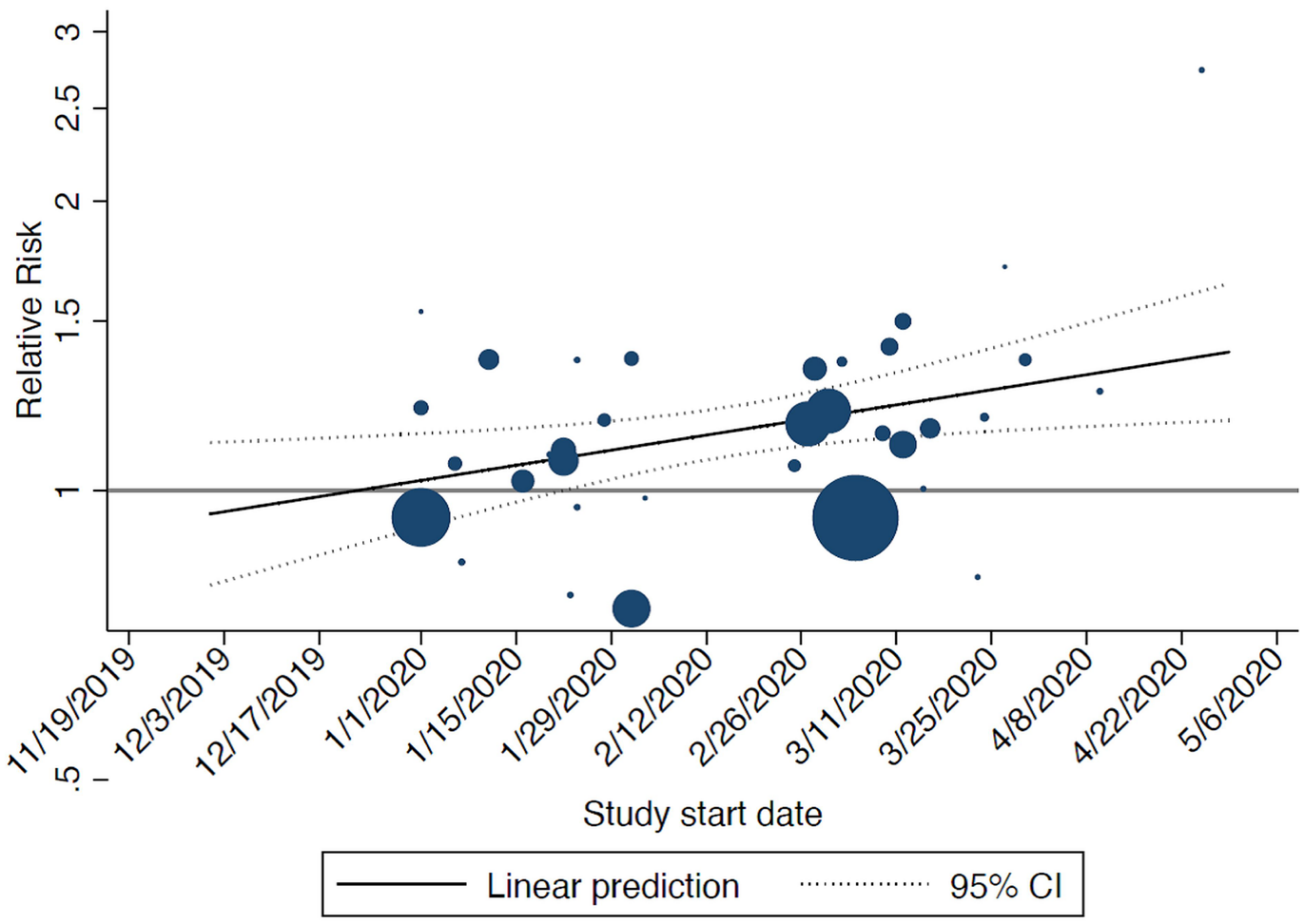
Bubble plot showing the results from the meta-regression on start date of included studies and relative risk of infection (men vs women): the later the start date of the study the higher the risk of infection for men compared to women. The size of the circles is inversely proportional to the variance of the estimated treatment effect. The dashed lines represent the limits of the 95% confidence interval (CI).

In total, 41 studies conducted in the general population reported data on the risk of infection and sex ( Table 2). Men were shown to have a higher risk of infection; the pooled RR from these studies was estimated to be 1.14 (95%CI: 1.07–1.21). Four studies from China, two studies from South Korea and United States each and one study from Thailand and United Kingdom each reported a higher risk of infection among women (see Figure appendix III in the *Extended data* ^12^). In the sensitivity analysis, the study start date was significantly associated with the relative risk of infection: the later the start date of the study the higher the risk of infection for men compared to women ( Figure 2).

**2. Hospitalisation**

Overall, 22 studies reported on hospitalisations among confirmed COVID-19 cases. When diagnosed, the risk of hospitalization was about 33% ( Table 2) more for men as compared to women (RR: 1.33, 95%CI: 1.27 – 1.41), (Figure appendix III in the *Extended data* ^12^)

**3. Disease severity**

**Figure 3.**
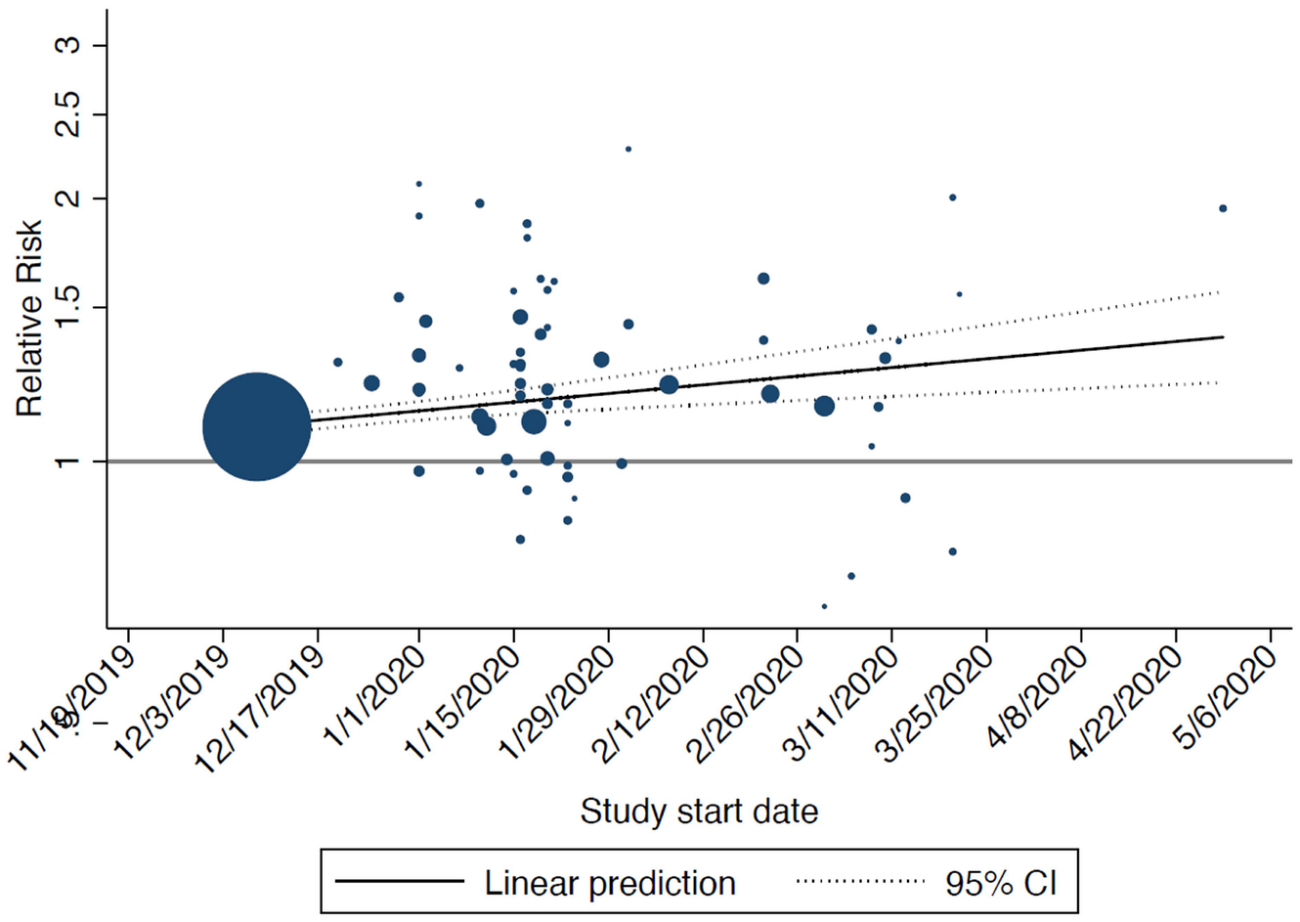
Bubble plot showing the results from the meta-regression on start date of included studies and relative risk of disease severity (men vs women): the later the start date of the study the higher the risk of severe of disease for men compared to women. The size of the circles is inversely proportional to the variance of the estimated treatment effect. The dashed lines represent the limits of the 95% confidence interval (CI).

In total, 77 studies reported on disease severity among populations hospitalised due to COVID-19. When hospitalised, men experienced more severe disease than women (RR = 1.22, 95%CI: 1.17- 1.27) implying that the risk of severe disease of COVID-19 for men is 22% higher than that for women ( Table 2, Figure appendix III in the *Extended data* ^12^). In the sensitivity analysis, start date was significantly associated with the relative risk disease severity: the later the start date of the study the higher the risk of disease severity for men compared to women ( Figure 3).

**4. ICU admittance**

**Figure 4.**
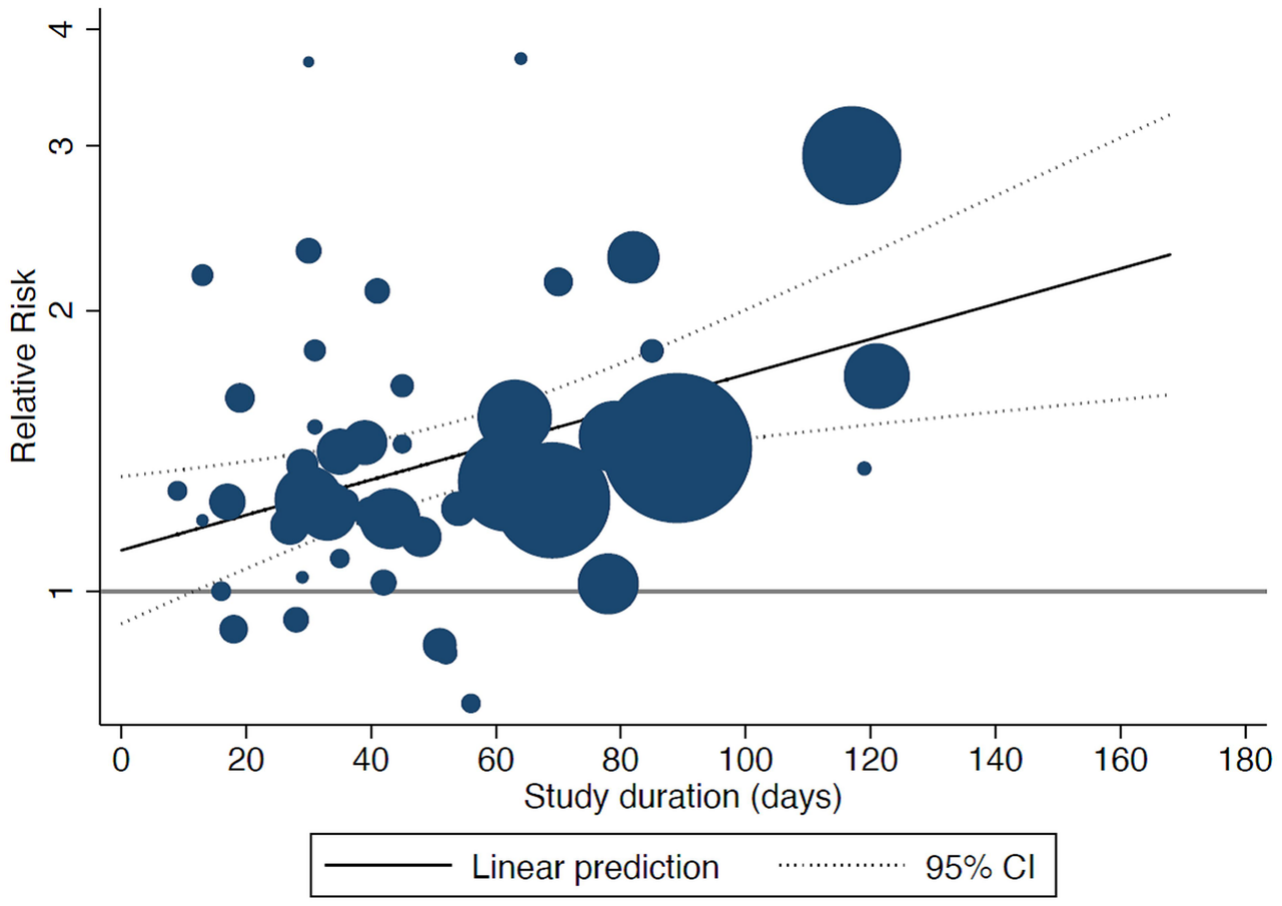
Bubble plot showing the results from the meta-regression on study duration of included studies and relative risk of intensive care unit (ICU) admission (men vs women): the longer the duration of the study, the higher the relative risk of admission to ICU for men than women. The size of the circles is inversely proportional to the variance of the estimated treatment effect. The dashed lines represent the limits of the 95% confidence interval (CI).

In total, 48 studies reported on ICU admittance among hospitalised COVID-19 cases. The rate of admission to ICU in COVID-19 hospitalised patients was higher among men as compared to women. The aggregated effect size was 1.41 with a 95%CI of 1.28–1.55 ( Table 2, Figure appendix III in the *Extended data* ^12^). In the sensitivity analysis, study duration was significantly associated with the relative risk of ICU admittance: the longer the duration of the study, the higher the relative risk of admission to ICU for men than women ( Figure 4).

**5. Death**

**Figure 5.**
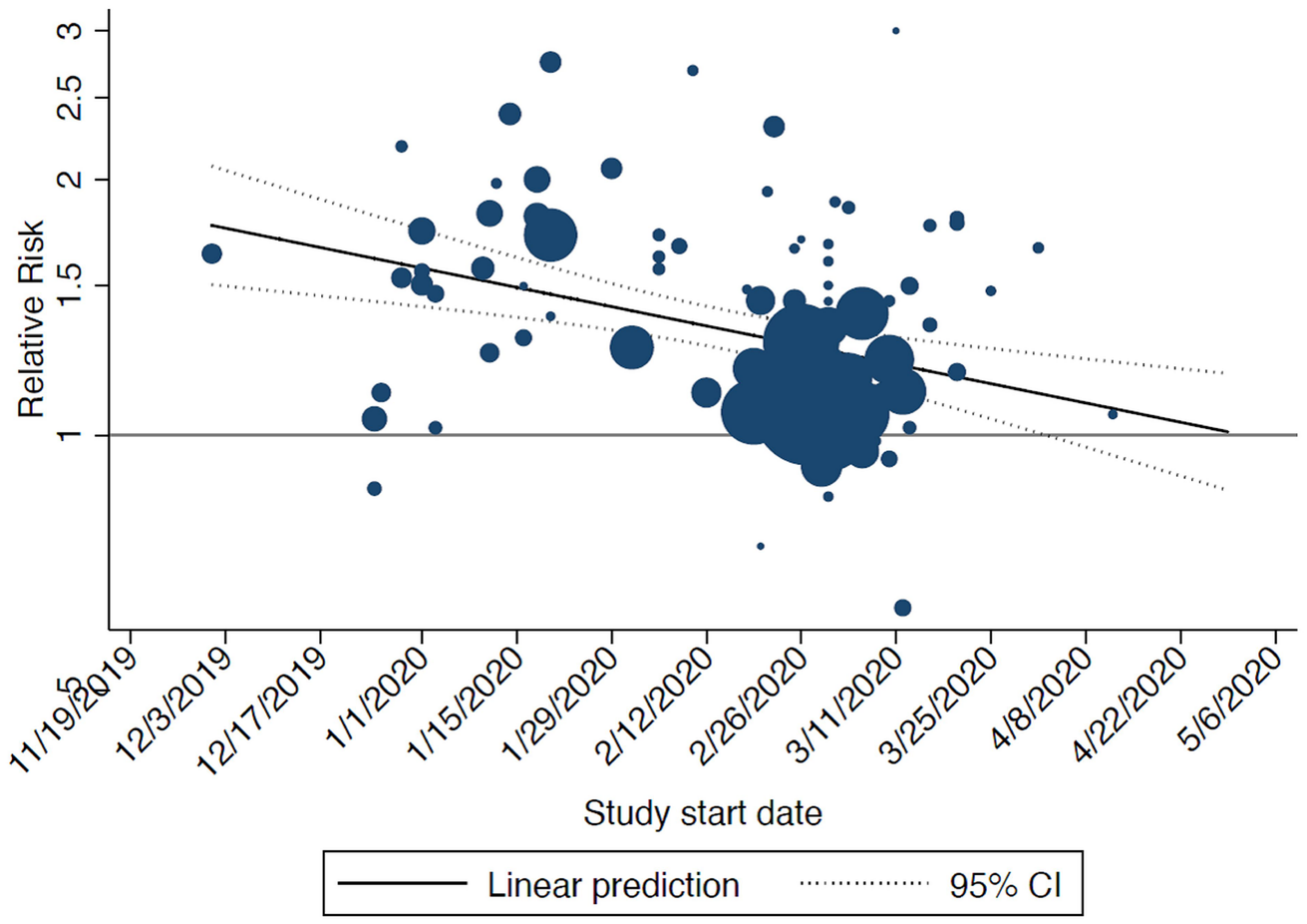
Bubble plot showing the results from the meta-regression on start date of included studies and relative risk of death (men vs women): the later the start date of the study the lower the risk of death for men compared to women. The size of the circles is inversely proportional to the variance of the estimated treatment effect. The dashed lines represent the limits of the 95% confidence interval (CI).

Overall, 91 studies reported on death among hospitalized COVID-19 cases. We observed that men were at higher risk of death ( Table 2, Figure appendix III in the *Extended data* ^12^) from COVID-19 as compared to women (RR = 1.35, 95%CI: 1.28–1.43). In the sensitivity analysis, start date was significantly associated with the relative risk of death: the later the start date of the study the lower the risk of death for men compared to women ( Figure 5).

###  Sensitivity analyses

**1. Overlap in study populations**

Considering the vast number of studies on COVID-19 the potential effect of overlap of study populations on the results should be evaluated. In instances where two studies were from the same region, had similar or overlapping patient recruitment periods or same hospitals- one could suspect a possible overlap in the studied populations. We conducted a sensitivity analysis by only including the largest study from a group of studies with a possible study population overlap.

Overall, the estimates from the meta-analysis were very robust and the results did not demonstrate any substantial shifts in pooled effect estimates for any of the five study outcomes after exclusion of studies with possible overlap ( Table 3).

**Table 3.** Exclusion of possible overlaps.

		All studies		Excluding possible overlap	
Exposure	Outcome	Number of studies	Pooled estimate (RR)	Number of studies	Pooled estimate (RR)
Sex (male vs female)	Infection	41	1.14	**28**	**1.18**
	Hospitalization	22	1.33	**18**	**1.35**
	Severe disease	77	1.22	**17**	**1.28**
	ICU	48	1.41	**37**	**1.39**
	Death	91	1.35	**40**	**1.27**

Studies with possible overlap of patients were excluded from the analysis, **results presented in bold**. Possible overlap was assumed when studies were from the same region, recruitment period and hospital. In a group of studies with possible overlap only the largest study was included in the analysis. The estimates from the meta-analysis were very robust and did not demonstrate any substantial shifts in pooled effect estimates for any of the five study outcomes after exclusion of studies with possible overlap. RR = relative risk; ICU=intensive care unit.

**2. Heterogeneity**

In our primary outcome analysis ( Table 2), we observed moderate to substantial heterogeneity. To address this, we also performed extensive sensitivity analyses consisting of subgroup and meta-regression analyses (Appendix IV in the *Extended data* ^12^). The conclusions of our study did not markedly change with subgroup analyses and meta-regression analyses.

**3. Publication bias**

Funnel plots showed some asymmetry for the relation between sex and the outcomes of severe disease and death (p-values of 0.000 and 0.030, respectively; Harbord test). Although the subsequent trim-and fill analysis revealed some reduction in the effect sizes, all conclusions remained the same. More specifically, the RR for severity changed from 1.22 to 1.19 and for death from 1.35 to 1.20. 

## Discussion

### Summary of evidence

In this systematic review, we evaluated the association between sex and COVID-19 infection, hospitalization, disease severity, ICU admission and death based on studies from across the world. Our results showed that men were more likely to be affected or to be more severely affected by COVID-19 than women on all disease outcomes. Men appear to have a higher risk of COVID-19 infection (as compared to women). When infected, they were observed to have a higher risk of hospitalization, and when hospitalized they had higher risk of severe COVID-19 disease and ICU admission, and ultimately a higher risk of dying. We also observed that, within the time period studied, the relative risk of infection and severe disease increased for men compared to women, while the relative risk of mortality decreased for men compared to women. Additionally, the relative risk of ICU admission increased for men compared to women when the duration of the study increased, suggesting that focus on ICU admission should have sufficient follow-up.

### Interpretation

The various hierarchical COVID-19 related outcomes were estimated among different populations, i.e. infection among the general population, hospitalization among people with confirmed infections, and severe disease, ICU admission and death among all hospitalized patients with COVID-19.

Within each of these domains, different mechanisms may be responsible for the observed associations of sex with these outcomes.

The risk of an individual for confirmed COVID-19 infection among the general population is the sum of several mechanisms, including the population spread in their area, their exposure to other people in occupational, leisure and social setting and the mitigating measures imposed by local government. These mechanisms are likely to play a different role across different geographical regions and the way they affect men and women differently. In some countries, working outside of the house with associated exposure is more equally divided between men and women than in other countries. The part of each sex in caring activities (e.g. child care or informal care for other family members) also likely varies between geographical regions and cultures. Indeed, we observed divergent risks that seem clustered within countries, most notably the higher risk of infection among women in two large studies in South Korea ^19, 20^. In South Korea, women were indeed over-represented in higher-risk professions i.e. among nurses, medical and welfare-related healthcare sectors; as well as social welfare-related sectors. Besides that, more women were employed in high-risk professions (like hairdressing, wedding service workers, household helpers, cooking attendants, educational professionals and related occupations) ^21^. In addition, sexes show difference on attitude and behaviour facing COVID-19, in that women are more likely to perceive COVID-19 as a very serious health problem, to agree with restraining public policy measures, and to comply with them ^22– 24^. It, therefore, might cause men with more chances to be exposed to SARS-CoV-2. Most other countries show a consistently higher risk of men for COVID-19 infections, although the USA shows inconsistent estimates across studies. This may be related to the governing context where measures and work and care cultures vary considerably across different states. A sensitivity analysis on the timeframe of the study showed a trend towards increasing risk for men over time.

The risk of hospitalization when infected with COVID-19 should be interpreted within both the medical and health care context. First, there may be medical mechanisms in play that cause men and women to divergently develop COVID-19 severity that requires hospitalization. Indeed, other respiratory tract infections tend to be more severe in men ^25^. However, hospital capacity was limited at times in some parts of the world due to peak admissions, possibly influencing the selection of patients that were admitted.

The risk of outcomes related to the severity of COVID-19 (i.e. severe disease, ICU admissions and death) among patients hospitalized for COVID-19 is considered to be more independent of societal context and relative risks of men compared to women should be interpreted as reflecting biological mechanisms. During the severe acute respiratory syndrome (SARS) epidemic of 2003 mortality was also higher in men ^26^. Over the course of the COVID-19 pandemic, clinical care and treatment have improved due to increased knowledge on the clinical manifestations and course of disease. It seems that absolute risks of dying among hospitalized patients, both within each sex and overall, have decreased over time ^7– 9^.

### Implications for clinicians, policymakers and researchers

All relative risks that have been presented in this meta-analysis are univariable associations between sex and COVID-19 outcomes. This means that, although men indeed experience higher rates on all outcomes (infection through death) in the real world, sex may not be the causal factor causing an increased risk at some outcomes. Instead, the observed associations may be the result of other causal factors which are unequally distributed between both sexes. Indeed, there is literature to support hypotheses of causal effects of comorbidities, weight distribution, and immunological characteristics. as mechanisms through which differences in outcomes are observed between sexes ^4– 6, 8^.

Nevertheless, when researchers, clinicians and policymakers want to target the appropriate group for interventions (healthcare or vaccinations) or studies, these findings based on real-world data may be helpful in identifying sub-populations or important confounders. Additionally, they may play a role in hypotheses generation regarding causal mechanisms of COVID-19 outcomes.

### Limitations and strengths

Some limitations of this study should be considered. In some studies on death, information on the subjects without an endpoint was missing, so there was a high risk of non-differential misclassification that could lead to bias

Another limitation is that although we have found that the RR for infection, disease severity and mortality changed during the course of the pandemic, our study is not designed to provide possible explanations. Future studies are required to explore possible mechanisms.

Our review has the following strengths. Our search strategy was thorough and complete: we screened 10,160 individual records in addition to 11,550 hits of the previous paper on the first wave ^2^. This meta-analysis includes papers based on worldwide and from various phases of the pandemic—from the first wave to the second wave before variants of concern were identified. Such variants of concern are likely to alter the overall absolute risk of several outcomes in this meta-analysis, and potentially affect relative risks of sexes for these outcomes as well. In the studies included, we have observed a large range of absolute risks as a result of studies from different phases of the pandemic and from various regions, to which the relative risk of sex seemed to be fairly robust. This is however not guaranteed for other strains and this possibility should be considered when we extrapolate these results to more recent settings.

Our search strategy therefore ensured that the included studies cover a wide range of transmission-mitigation policies, cultures and clinical settings and that the included studies cover a mix of virus strains that are (clinically) similar in terms of transmissibility and disease severity. The methodological quality as reflected by the NOS score was high and a thorough sensitivity analysis could not refute the conclusions. The possible influence of publication bias was considered to be small as only small changes in effect size after the trim-and-fill analyses were observed.

## Conclusion

We systematically reviewed the literature to determine the relation between sex as a risk factor for COVID-19 infection, hospitalisation, disease severity, ICU admission and death. Meta-analyses on 229 studies comprising 10 million patients showed that men have a higher risk for infection, hospitalisation, severe disease, ICU admission and death. Within the period studied, the relative risk for infection and severe disease increased for men compared to women, while the relative risk for mortality decreased for men compared to women.

## Data availability

### Underlying data

Harvard dataverse: Replication Data for: Temporal trends of sex differences for COVID-19 infection, hospitalisation, severe disease, intensive care unit (ICU) admission and death: Aa meta-analysis of 229 studies covering over 10M patients. https://doi.org/10.7910/DVN/DPP67G ^18^.

### Extended data

Harvard Dataverse: Appendix for: Temporal trends of sex differences for COVID-19 infection, hospitalisation, severe disease, intensive care unit (ICU) admission and death: Aa meta-analysis of 229 studies covering over 10M patients. https://doi.org/10.7910/DVN/JPLKI9 ^12^.

### Reporting guidelines

Harvard Dataverse: PRISMA checklist for ‘Temporal trends of sex differences for COVID-19 infection, hospitalisation, severe disease, intensive care unit (ICU) admission and death: a meta-analysis of 229 studies covering over 10M patients’ https://doi.org/10.7910/DVN/TRIZJX ^10^.

Data are available under the terms of the Creative Commons Zero "No rights reserved" data waiver (CC0 1.0 Public domain dedication).

## Transparency declaration

The manuscript’s guarantors (BP, SJ and MZ) affirm that the manuscript is an honest, accurate, and transparent account of the study being reported; that no important aspects of the study have been omitted; and that any discrepancies from the study as planned (and, if relevant, registered) have been explained.
