# Supplementary material for: Temporal trends of sex differences for COVID-19 infection, hospitalisation, severe disease, intensive care unit (ICU) admission and death: a meta-analysis of 229 studies covering over 10M patients
Source: F1000Res. 2022 Jan 5;11:5. [Version 1] doi: 10.12688/f1000research.74645.1 (PMC9034173; doi:10.12688/f1000research.74645.1)
Supplement: Peer review history

# Peer review: Reviewer response for version 1

**DOI:** 10.5256/f1000research.78420.r129830

**Published:** 2022-04-22

**Authors:** Helder I. Nakaya

**Affiliations:** 1 University of São Paulo, São Paulo, Brazil

Pijls *et al* provided a comprehensive meta-analysis that shows the temporal trends of sex differences for different COVID-19 outcomes. The study selection, the meta-analysis itself (following PRISMA), statistical analysis, and conclusions are appropriate. However, the main conclusion (men are more infected and have an increased risk of severe COVID-19 than women) was shown by several people. It is not clear whether the authors had access to demographic and clinical anonymized information of all 10M+ patients. If so, this data should be provided.

 As studies themselves have different characteristics regarding the recruited patients (comorbidities, age group, etc), I wish the authors had focused on trying to explain which conditions these relative risks are much higher or much lower in men. Also, (as stated in the "limitations and strengths") this manuscript only contains data from the first wave of COVID-19 mostly. It would be interesting to see these risks over the different waves and variants.

Are the rationale for, and objectives of, the Systematic Review clearly stated?

Yes

Is the statistical analysis and its interpretation appropriate?

Yes

Are sufficient details of the methods and analysis provided to allow replication by others?

Yes

Are the conclusions drawn adequately supported by the results presented in the review?

Yes

Reviewer Expertise:

Data science, bioinformatics, immunology, systems biology

I confirm that I have read this submission and believe that I have an appropriate level of expertise to confirm that it is of an acceptable scientific standard.

# Peer review: Reviewer response for version 1

**DOI:** 10.5256/f1000research.78420.r125485

**Published:** 2022-03-23

**Authors:** Kevin Wing

**Affiliations:** 1 London School of Hygiene and Tropical Medicine, London, UK

In this large and comprehensive systematic review and meta-analysis, the authors looked at the association between sex and COVID-19 health outcomes, screening over 10 000 studies in six different languages from searches of PubMed and EMBASE (between December 2019 and September 2020). Pooled relative risks for 229 selected studies were determined using a random-effects meta-analysis, with a number of factors investigated as potential sources of heterogeneity (including geographical region and time from start of pandemic). Men were shown to have a higher risk for all outcomes overall, with a lower risk for infection and severity of disease and higher risk for death earlier in the pandemic.

 I thought this was an impressive and valuable piece of research, with only a number of minor suggestions for edits, as follows:

1. Abstract - it would be helpful to include the start date of the search
2. In the first paragraph of the Introduction, please provide a date that supports the phrase "early phase of the pandemic" (e.g "(prior to May 2020)")
3.  In Table 1, there are columns of "case" and "control", which suggests case control studies, although from the underlying data it looks like there were very few of these. I would recommend removing the "control" column completely and renaming the "Case" column "Outcome (case)"
4. Methods - Study selection section: please rephrase "Studies were excluded when there was no data on controls" to read "Studies were excluded if they only included people with the outcome (cases)".
5. Methods - data synthesis and analysis: I was surprised to see that study design (e.g. cohort vs case control vs cross sectional) wasn't included as a subgroup - if numbers of non-cohort studies are a problem then this could be cohort vs other designs (see also comment re: South Korean studies in interpretation).
6. Results - Study selection section, please rephrase to "49 only included cases"
7. Results - Study characteristics, please add a sentence describing the study designs (e.g. "X studies were cohort designs, with Y cross-sectional and Z case control").
8. Results - In Table 2, the "Number of patients" column is blank.
9. Appendix Forest Plots - the format of the plots for Infection, Hospitalization, and ICU admission (vertical forest plots with countries shown) is much more informative than the plots for Severe Disease and Death - could these two be updated to match the others?
10. In the results and appendices  "sensitivity analysis" are referred to but this term is not used in the methods. I think what is being referred to here is the results of "subgroup (and meta-regression) analysis" - it would help if this terminology was used instead of "sensitivity analysis" throughout (or "subgroup (and meta-regression) analysis" when it first appears, defining that this will be referred to as "subgroup analyses" subsequently).
11. In the Summary of Evidence section, I did not understand the text after the comma in the sentence "Additionally, the relative risk of ICU admission increased for men compared to women when the duration of the study increased, suggesting that focus on ICU admission should have sufficient follow-up."
12.  In Interpretation, re: the South Korean studies:
  a) could you also comment on whether the two South Korean studies were performed at an early stage of the pandemic i.e. is the early-pandemic effect that you have found also contributing here?
  b) I notice that neither of the South Korean studies are cohort studies (one is cross sectional, the other case control) but non-cohort studies were not investigated as a subgroup.
13. Implication for clinicians, policymakers, and researchers: I wasn't sure about the statement "All relative risks that have been presented in this meta-analysis are univariable associations between sex and COVID-19 outcomes" as it is not specified anywhere in the methods that only unadjusted effects were being pooled. Could this be clarified?

Are the rationale for, and objectives of, the Systematic Review clearly stated?

Yes

Is the statistical analysis and its interpretation appropriate?

Yes

Are sufficient details of the methods and analysis provided to allow replication by others?

Yes

Are the conclusions drawn adequately supported by the results presented in the review?

Yes

Reviewer Expertise:

Epidemiologist with experience of performing analysis of COVID outcomes in very large datasets and of performing systematic reviews.

I confirm that I have read this submission and believe that I have an appropriate level of expertise to confirm that it is of an acceptable scientific standard.
